# Progress in phase III clinical trials of molecular targeted therapy and immunotherapy for glioblastoma

**DOI:** 10.1002/cai2.59

**Published:** 2023-03-05

**Authors:** Yuekun Wang, Shenglan Li, Yichen Peng, Wenbin Ma, Yu Wang, Wenbin Li

**Affiliations:** ^1^ Department of Neurosurgery, Peking Union Medical College Hospital Chinese Academy of Medical Sciences and Peking Union Medical College Beijing China; ^2^ Department of Neuro‐oncology, Cancer Center, Beijing Tiantan Hospital Capital Medical University Beijing China

**Keywords:** glioblastoma, immunotherapy, phase III clinical trial, target therapy

## Abstract

Glioblastoma (GBM) is the most common primary central nervous system tumor, whose prognosis remains poor under the sequential standard of care, such as neurosurgery followed by concurrent temozolomide radiochemotherapy and adjuvant temozolomide chemotherapy in the presence or absence of tumor treating fields. Accordingly, the advent of molecular targeted therapy and immunotherapy has opened a new era of tumor management. A diverse range of targeted drugs have been tested in patients with GBM in phase III clinical trials. However, these drugs are ineffective for all patients, as evidenced by the fact that only a minority of patients in these trials showed prolonged survival. Furthermore, there are several published phase III clinical trials that involve immune checkpoint inhibitors, peptide vaccines, dendritic cell vaccines, and virotherapy. Accordingly, this review comprehensively overviews existing studies of targeted drugs and immunotherapy for glioma and discusses the challenge and perspective of targeted drugs and immunotherapy for glioma to clarify future directions.

## Graphical abstract

A wide variety of targeted drugs and immunotherapies are tested for patients with glioblastoma, but not all of these drugs are effective, and only a few of these trials have prolonged the lives of patients. We summarize the phase III clinical trial of target therapy and inmmunotherapy for glioblastoma and discuss the challenge faced contemporaneously and prospects of targeted drugs and immunotherapy in the future.

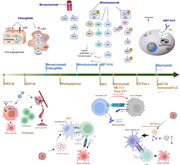

**Abbreviations**
5‐FC: 5‐fluorocytosine
5‐FU: 5‐fluorouracil
AKTp: protein kinase B
BBB: blood–brain barrier
CAR‐T: chimeric antigen receptor T‐cell immunotherapy
CCNU: lomustine
CNS: central nervous system
CTL: cytotoxic T lymphocytes
DC: dendritic cell
EGFR: epidermal growth factor receptor
EGFR: epidermal growth factor receptor
ER: endoplasmic reticulum
ERK: extracellular regulated protein kinases
GBM: glioblastoma
HLA: human leukocyte antigen
ICIs: immune checkpoint inhibitors
IDH: isocitrate dehydrogenase
ITT: Intention‐to‐Treat
MAPK: mitogen‐activated protein kinase
MGMT: *O*‐6‐methylguanine‐DNA methyltransferase
MMR: mismatch repair
mOS: median overall survival
mTOR: mammalian target of rapamycin
NCCN: National Comprehensive Cancer Network
PD‐1: programmed cell death protein 1
PD‐L1: programmed cell death 1 ligand 1
PFS: progression‐free survival
PPE‐1: pre‐proendothelin 1
PPV: personalized peptide vaccination
TERT: telomerase reverse transcriptase
TMB: tumor mutational burden
TMZ: temozolomide
TNFR1: tumor necrosis factor receptor 1
TTF: tumor treatment fields
VEGF: vascular endothelial growth factor
VEGFR: vascular endothelial growth factor receptor

## 1 INTRODUCTION

Glioblastoma (GBM), the most prevalent primary malignant brain tumor, is associated with a dismal prognosis and poor quality of life [1, 2, 3, 4]. In addition, this tumor has an annual morbidity rate of 3.26 per 100,000 [5]. GBM usually recurs even after standard therapy, and it is estimated that only an average of 6–11 months elapses between tumor recurrence and progression [6]. The World Health Organization (WHO) classification of glioma has been rapidly modified since molecular alternation was added to the third edition in 2000 and continually changed in the latest fifth edition in 2021 [7, 8, 9]. Based on the fifth edition of the WHO Classification of Tumors of the Central Nervous System (WHO CNS5), diffuse astrocytic glioma with wild‐type (WT) isocitrate dehydrogenase (IDH) and WT Histone 3 (H3) should be diagnosed as “IDH‐WT GBM, WHO grade 4” in the presence of at least one of the following pathological and genetic features, including (1) microvascular proliferation; (2) necrosis; (3) telomerase reverse transcriptase (TERT) promoter mutation; (4) Epidermal Growth Factor Receptor (EGFR) gene amplification; and (5) chromosome 7 gain‐of‐function and chromosome 10 loss‐of‐function copy number variants. The previous diagnosis of “IDH‐mutant GBM” should be corrected to “IDH‐mutant astrocytoma, WHO grade 4” in the presence of at least one of the following features: microvascular proliferation, necrosis, and homogeneous deletion of the CDKN2A/B gene [10].

Treatment of GBM is complicated by multiple factors, including tumor heterogeneity within and between patients and a highly impermeable blood–brain barrier (BBB), which limits the effectiveness of numerous standard therapies. Currently, GBM patients still have a disappointing prognosis even when receiving the standard of care, including maximal surgical excision followed by temozolomide (TMZ) radiochemotherapy and adjuvant TMZ chemotherapy with/without tumor treating fields (TTF), as evidenced by a median overall survival (mOS) of 20.5 and 15.6 months, respectively [11]. Of note, advances in molecular targeted therapy and immunotherapy have started a new era of GBM treatment. National Comprehensive Cancer Network guidelines recommend clinical trials as a good practical point for research on GBM. Therefore, we reviewed the phase III clinical trials of molecular targeted therapy and immunotherapy for GBM (Tables 1 and  2) and discussed the contemporary challenges and future landscape of these two therapies. For most of these trials, GBM was diagnosed with the fourth edition WHO classification of Tumors of the Central Nervous System, and others were based on pathological diagnoses. Post hoc analysis of previous clinical trials and prospective studies based on WHO CNS5 are needed.

**Table 1:** Phase III clinical trial of molecular targeted therapy for high‐grade glioma.

Gene‐features targets	Number	Treatment	Type of study	Setting	No. of patients	Outcome or conclusion	Ref.
IDH1/2	NCT00753246	Nimotuzumab	An open label, randomized phase III study	GBM	149	Negative	[12]
	NCT02573324	ABT‐414	A randomized, placebo‐controlled phase III study	Newly diagnosed glioblastoma (GBM) with EGFR amplification	655	Ongoing	[13]
	NCT01480479	Rindopepimut	A randomized phase III, placebo‐controlled	Newly diagnosed glioblastoma confirmed to express EGFRvIII	745	mPFS (months): rindopepimut 7.1, placebo 5.6	[14]
						mOS (months): rindopepimut 20.1, placebo 20.0	
Microenvironmental targets							
VEGFR	NCT00884741	Bevacizumab	Phase III	Newly diagnosed GBM	637	mPFS:	[15]
						Bevacizumab+ 10.7 months	
						Bevacizumab− 7.3 months	
						mOS:	
						Bevacizumab+ 15.7 months	
						Bevacizumab− 16.1 months	
	NCT00943826	Bevacizumab	Phase III	Newly diagnosed GBM	349	IDH1 wild‐type proneural GBM may have an OS benefit from Bevacizumab	[16]
	NCT00689221	Cilengitide	Phase III, Open Label	Newly diagnosed glioblastoma and methylated gene promoter status	545	mOS:	[17, 18]
						Cilengitide 26.3 (23.8–28.8)	
						Control 26.3 (23.9–34.7)	
						αvβ3 expression+MGMT promoter unmethylation may benefit from integrin inhibition	
Other potential targets	NCT03345095	Marizomib	Phase III	Newly diagnosed GBM	749	Ongoing	

Abbreviations: GBM, glioblastoma; mOS, median overall survival; mPFS, mdian progression‐free survival; VEGFR, vascular endothelial growth factor receptor.

**Table 2:** Summarization of published phase III clinical trials of immunotherapy of GBM.

Reference	Published year	Immunotherapy	Population	Size of interventional group	Intervention	Size of control group	Control	Results
Reardon et al. [19]	2020	ICIs	rGBM	184	NIVO	185	Bevacizumab	mOS: 13.4 versus 14.9 m
								mPFS: 6.0 versus 6.2 m
								3–4 grades AE: 21.9% versus 25.1%
Omuro et al. [20]	2022	ICIs	nGBM, MGMT promoter unmethylation	280	RT+NIVO, adjuvant NIVO	280	STUPP regimen	mOS: 28.9 versus 32.1 m
								mPFS: 10.6 versus 10.3 m
								3–4 grades AE: 18.1% versus 15.2%
Lim et al. [21]	2022	ICIs	nGBM, MGMT promoter methylation	358	RT+TMZ+NIVO, adjuvant TMZ+NIVO	358	STUPP regimen+placebo	mOS: 9.8 versus 10.0 m
								mPFS: 1.5 versus 3.5 m
								3–4 grades AE: 52.4% versus 33.6%
Weller et al. [22]	2017	Peptide vaccine	nGBM	369	Rindopepimut+TMZ	372	Keyhole hemocyanin+TMZ	MRD
								mOS: 19.5 versus 19.2 m
								mPFS: 8.0 m versus7.4 m
								SRD
								mOS: 16.1 versus 15.6 m
								mPFS: 3.7 versus 3.7 m
Narita et al. [23]	2019	Peptide vaccine	rGBM	58	PPV	30	Placebo	mOS: 8.4 versus 8.0 m
Liau et al. [24]	2022	DC vaccine	nGBM	232	DCVax‐L+TMZ	1366	Historical control	mOS: 19.3 versus 16.5 m
			rGBM	64	DCVax‐L	640	Historical control	mOS: 13.2 versus 7.8 m
Rainov [25]	2000	Virotherapy	nGBM	124	Surgery+RT+HSV‐tk+Ganciclovir	124	Surgery+RT	mOS: 365 versus 354 d
Westphal et al. [26]	2013	Virotherapy	nHGG	119	SOC+ADV‐tk+Ganciclovir	117	SOC	mOS: 497 versus 452 d
Cloughesy et al. [27]	2020	Virotherapy	rGBM	128	VB‐111+Bevacizumab	128	Bevacizumab	mOS: 6.8 versus 7.9 m
								mPFS: 3.4 versus 3.7 m
								≥3 grades AE: 67.5% versus 39.6%
Timothy F. Cloughesy [91]	2020	Virotherapy	rHGG	201	Toca 511+Toca FC	199	SOC (Lomustine/TMZ/Bevacizumab)	mOS:11.1 versus 12.2 m

Abbreviations: AE, adverse effects; DC, Dendritic cell; ICIs, immune checkpoint inhibitors; MGMT, *O*‐6‐methylguanine‐DNA methyltransferase; mOS, median overall survival; mPFS, mdian progression‐free survival; MRD, minimal residual disease; nGBM, newly diagnosed glioblastoma; nHGG, newly diagnosed high‐grade glioma; NIVO, nivolumab; PPV, personalized peptide vaccination; rGBM, recurrent glioblastoma; rHGG, recurrent high‐grade glioma; RT, radiotherapy; SOC, standard of care; SRD, significant residual disease; TMZ, temozolomide.

## 2 MOLECULAR TARGETED THERAPY

Because of the heterogeneity of GBM, key mutations related to GBM pathogenesis are being elucidated for the development of targeted therapies to effectively combat the complexity of GBM. In the following review, we will concisely analyze the progress in phase III clinical trials of molecular targeted therapy for GBM. As reported, most tumors involve recurrent molecular alterations that can block core growth pathways, such as mitogen‐activated protein kinase (MAPK), receptor tyrosine kinase (RTK), and phosphoinositide 3‐kinase (PI3K) pathways, cell cycle, DNA repair, and apoptosis.

### 2.1 Monoclonal antibodies

The vascular endothelial growth factor receptor (VEGFR) pathway has been widely recognized as a key factor in the survival of GBM cells [28]. Moreover, VEGF is overexpressed in GBM, which allows for the involvement of many downstream pathways, including MAPK/ERK1/2, endothelial nitric oxide synthase, and mTOR, in the abnormal proliferation of tumor vessels [29]. Accordingly, vascular proliferation is abnormal in GBM, while the normalization of vessels can increase tumor blood flow and improve the survival of GBM patients (NCT00305656) [30]. Of note, bevacizumab is a humanized monoclonal antibody against the VEGF‐A ligand, which has been reported to inhibit tumor angiogenesis [31]. Moreover, bevacizumab has been confirmed to elevate the survival of GBM patients. For example, a phase III trial revealed that bevacizumab significantly improved progression‐free survival (PFS) but insignificantly affected overall survival (OS) for patients with newly diagnosed GBM (nGBM) and recurrent GBM (rGBM) (NCT00884741) [15, 32]. Bevacizumab also prolongs the OS of patients with IDH1‐wt GBM (NCT00943826) [16]. The combination of bevacizumab and TMZ showed excellent efficacy and tolerability in patients with recurrent/progressing GBM [33]. In addition, bevacizumab combined with lomustine and radiotherapy also alleviated PFS in patients with IGS‐18 or “classical” GBMs [34].

### 2.2 The mammalian target of rapamycin (mTOR) inhibitor

Everolimus is an inhibitor of mTOR. According to a phase III trial, this inhibitor is effective in reducing the volume of subependymal giant cell astrocytomas [35]. Conversely, several studies have unveiled the disappointing results of mTOR inhibition on the OS of GBM patients [36].

### 2.3 Small‐molecule inhibitors

The efficacy of small‐molecule inhibitors targeting RTKs, particularly EGFR, has been extensively studied in GBM. EGFR amplification is found in 50% of cases, of which approximately half have EGFRvIII mutations [37]. EGFR inhibitors have not been manifested to improve survival in GBM patients despite their success in other cancers [38]. This phenomenon is attributed to relatively low intratumoral drug levels. Furthermore, the molecular heterogeneity of GBM and the simultaneous activation of multiple RTK pathways may also limit the efficacy of single target therapies [39]. EGFR antibodies have mostly failed in clinical trials for the treatment of gliomas [40, 41]. Intriguingly, nimotuzumab, an anti‐EGFR monoclonal antibody, is more effective in GBM patients with the activated AKT/mTOR pathway [12]. In addition, depatuxizumab mafodotin, an EGFR‐targeting antibody–drug conjugate, is effective in the treatment of rGBM that relapses after TMZ standard treatment [42] but is ineffective in the treatment of nGBM [13] (NCT02573324). The vaccine rindopepimut, in combination with TMZ demonstrated efficacy in response to rGBM with EGFRvIII mutations [43] (NCT00458601) but failed to show efficacy in a phase III clinical trial [44] (NCT01480479). Nonetheless, a small percentage of GBMs harbor driver mutations, such as BRAF V600E, that responds to RAF or RAF/MEK inhibitors or oncogenic fusions, such as NTRK [45, 46, 47].

The use of small molecules with multitarget inhibition may overcome issues, including heterogeneity and pathway redundancy, but may also increase toxicity. Regorafenib, an oral multikinase inhibitor, was found in a clinical trial to increase OS in patients with recurrent diseases, motivating further research [48]. Additionally, the effect of regorafenib on nGBM and rGBM is being evaluated in GBM AGILE, an international phase II/III trial designed to evaluate multiple treatment combinations (NCT03970447) [49].

### 2.4 Integrins

Integrins are a family of cell surface receptors composed of 24 types of heterodimers. Integrin signal transduction not only is involved in many cellular processes but also mediates cell communication within the extracellular matrix during adhesion, motility, migration, invasion, and angiogenesis. Moreover, prior studies indicated that integrin αvβ3 and αvβ5 were highly expressed in gliomas and were potential preclinical therapeutic targets for GBM [50, 51].

Cilengitide, a selective integrin inhibitor targeting αvβ3 and αvβ5, exhibits excellent tolerability and moderate efficacy in the treatment of rGBM as shown by clinical trials [52, 53, 54] (NCT00979862). In contrast, cilengitide cannot protect against invasion and improve recurrence in nGBM [55, 56]. Moreover, it has been reported that GBM patients with *O*‐6‐methylguanine‐DNA methyltransferase (MGMT) promoter methylation respond more favorably to cilengitide treatment than those with MGMT promoter unmethylation [17, 57] (NCT00689221). On the contrary, a phase III trial demonstrated that the efficacy of cilengitide is insignificant for GBM patients with MGMT promoter methylation [58] (NCT00689221). Despite the current dismal efficacy of targeting integrins for glioma treatment, this strategy has an excellent tolerability profile. Additionally, integrins are specifically expressed in tumors. Therefore, targeting integrins remains one of the most important research targets.

### 2.5 Targeting the proteasome

The proteasome, an intracellular protein degradation site [59], induces cancer cell apoptosis by regulating p53, ER stress, cell cycle, and drug resistance [60]. Combination of bortezomib (a proteasome inhibitor) and Vorinostat (a histone deacetylase inhibitor) is ineffective for the treatment of rGBM [61] (NCT00641706). Nevertheless, bortezomib combined with standard radiotherapy is safe and effective for nGBM patients [62] (NCT00998010). A phase I/II trial revealed that the proteasome inhibitor Marizomib in combination with bevacizumab is ineffective in the treatment of GBM patients [63] (NCT02330562). In addition, the combination of Marizomib and TMZ for GBM is in phase III trials (NCT03345095). The phase III clinical trials of molecular targeted therapy for GBM are listed in Table 1.

### 2.6 Antibody–drug conjugate (ADC)

An ADC, a combination of an antibody and a cytotoxic compound, can be used for the targeted delivery of biologically active molecules. Phase II trial results showed that the survival of patients with EGFR‐amplified rGBM was prolonged when Depatux‐M was combined with TMZ (INTELLANCE 2). However, a phase III trial of combination of Depatux‐M and TMZ for nGBM (INTELLANCE 1) was terminated because the interim analysis demonstrated no survival benefit (NCT02573324) [64].

### 2.7 Poly‐(ADP‐Ribose)‐DNA polymerase (PARP) inhibitors

**Figure 1:**
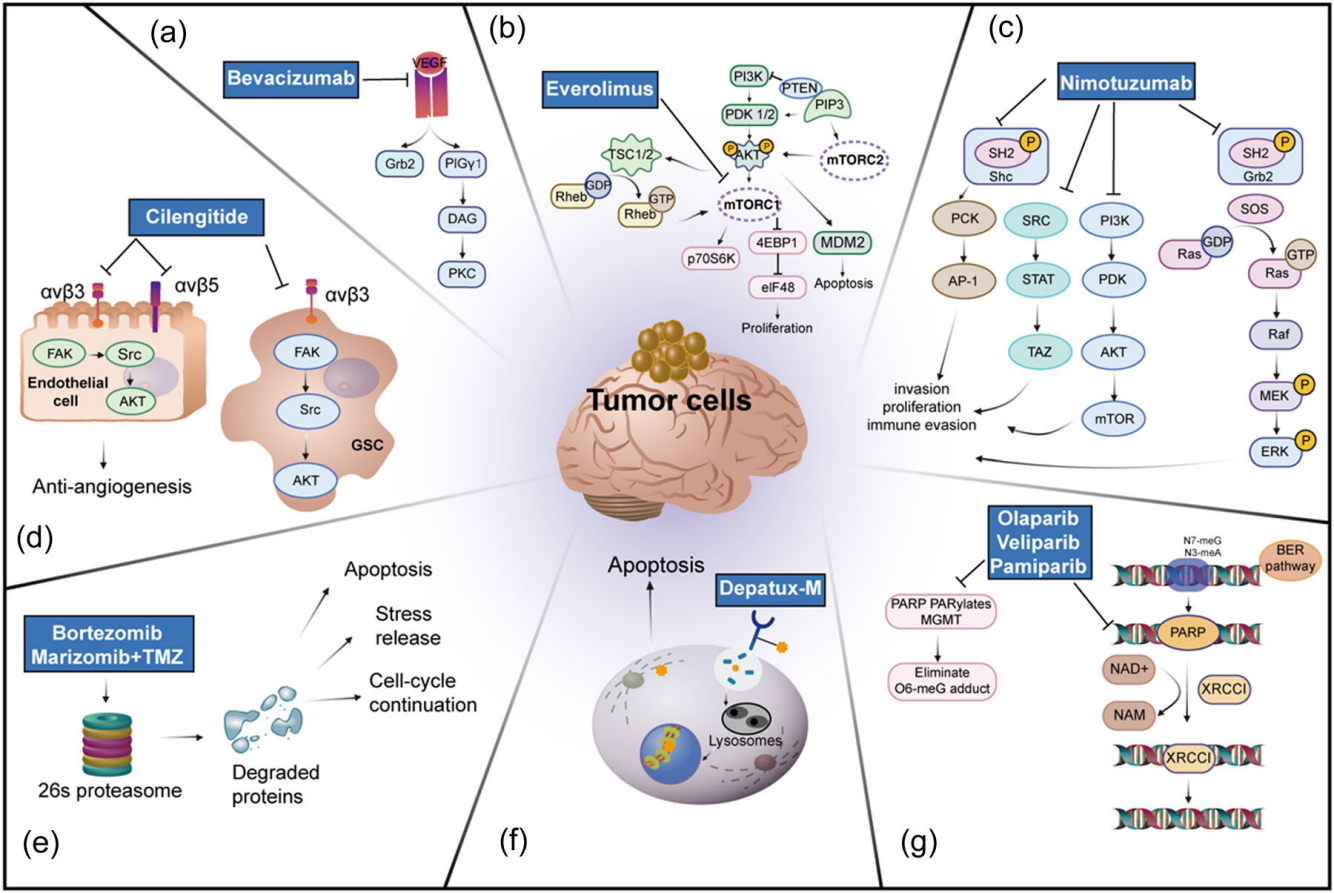
Overview of the mechanisms of different molecular targeted therapies in clinical trials of GBM. (a) Monoclonal antibodies used for the treatment of GBM. (b) Therapeutic agents targeting mTOR pathways for the treatment of GBM. (c) The functional mechanisms of the related small‐molecule inhibitors advanced in clinical trials of GBM. (d) Integrins used in the clinical trial of GBM. (e) Role of proteasome inhibitors in GBM. (f) ADC application in the treatment of GBM and its targets and effect. (g) PARP inhibitors for the treatment of GBM.

The efficacy of several PARP inhibitors against GBM has rarely been evaluated in the clinic. However, recent studies have illustrated that olaparib, veliparib, and pamiparib can achieve therapeutic levels in situ [65, 66, 67]. A phase III trial is underway to evaluate the combination of Veliparib and adjuvant TMZ for MGMT‐methylated nGBM, and the results will be available soon (NCT02152982). Mechanisms underlying different molecular targeted therapies for GBM in clinical trials are detailed in Figure 1.

## 3 IMMUNOTHERAPY

### 3.1 Immune environment of GBM

**Figure 2:**
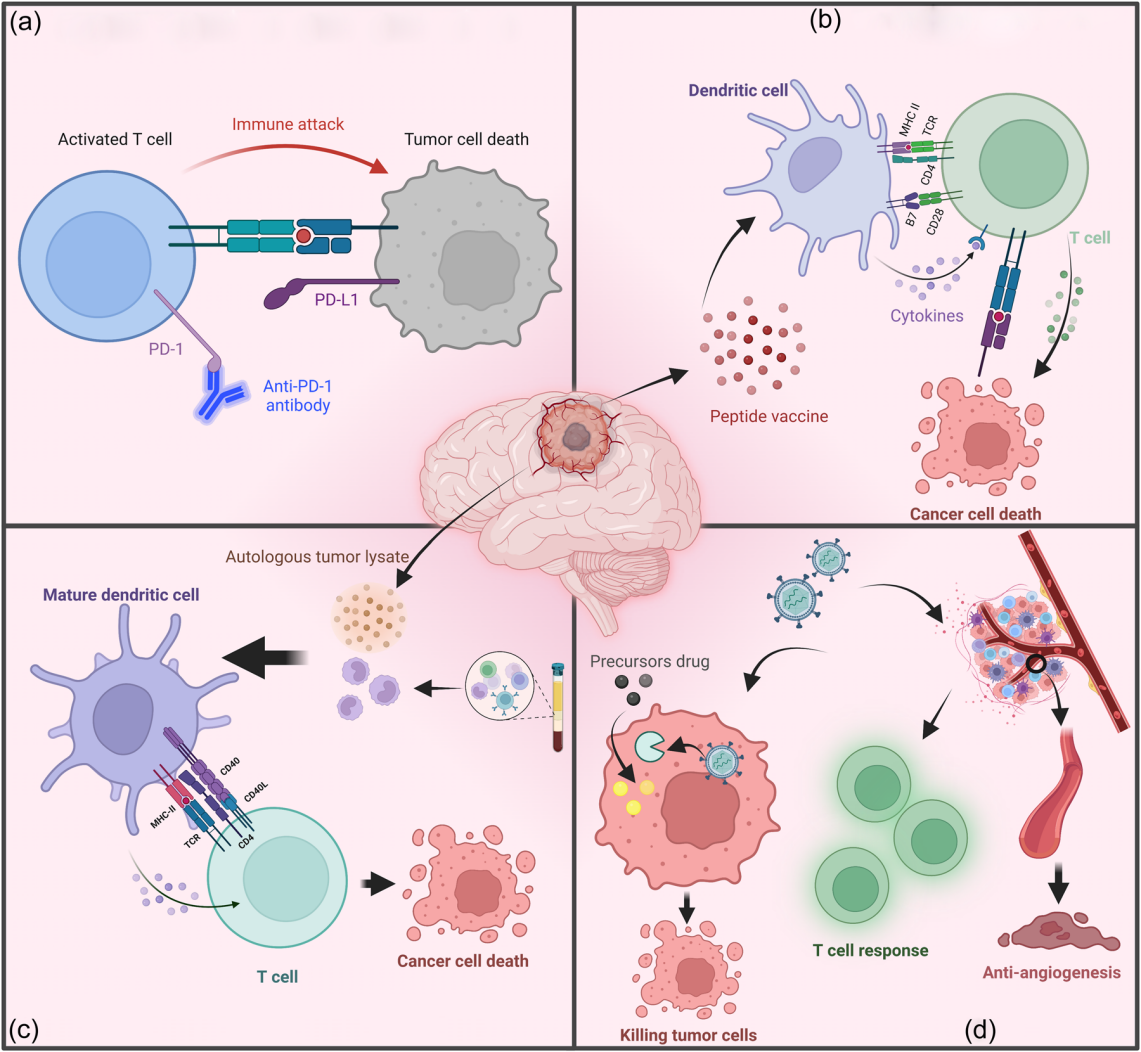
Brief illustration of immunotherapy for glioblastoma (GBM) whose phase III clinical trials finished. (a) Immune checkpoint inhibitors (anti‐PD‐1). (b) Antigenic peptide vaccine. (c) Dendritic cell vaccines (DCVax‐L). (d) Virotherapy.

The lymphatic vessels lining the dural sinus in the CNS were found in a mouse model, which can drain cerebrospinal fluids and lymphocytes to deep cervical lymph nodes. This finding changes the previous perception of the brain as an “immune‐privilege” organ and also provides support for immunotherapy of brain tumors [68]. GBM is characterized by a “cold” immune microenvironment with a relatively low somatic tumor mutational burden (TMB) and T lymphocyte infiltration [69]. The BBB, unique lymphatic system, and regulation of immune components are responsible for an immunosuppressive microenvironment, which challenges immunotherapy of GBM [70]. Mechanisms of GBM immunotherapy involved in phase III clinical trials are shown in Figure 2.

### 3.2 Phase III clinical trials of immunotherapy for GBM

#### 3.2.1 Immune checkpoint inhibitors (ICIs)

ICIs induce antitumor immune responses by targeting immunosuppressive pathways. CheckMate 143 [19] is the first phase III randomized clinical trial of ICIs for GBM, which involves Nivolumab and bevacizumab monotherapy (the control). The results exhibited that the mOS of patients was 9.8 and 10.0 months in the Nivolumab and bevacizumab groups, respectively (*p* = 0.76), and that median PFS (mPFS) was markedly longer in the bevacizumab group. Grade 3–4 adverse reactions in the Nivolumab group were mainly malaise, elevated liver enzymes, and elevated lipase. Omuro et al. conducted CheckMate 498 for newly diagnosed supratentorial GBM with MGMT promoter unmethylation in 2022 [20] and evaluated the efficacy of concurrent radiotherapy with Nivolumab followed by adjuvant Nivolumab by comparisons with standard STUPP regimen [71]. The results revealed that mOS was 13.4 and 14.9 months in the Nivolumab and STUPP groups, respectively (*p* = 0.0037). CheckMate 548 was also performed in 2022 for nGBM with MGMT promoter methylation [21], where patients in the Nivolumab group were treated with concurrent TMZ radiochemotherapy with adjuvant TMZ plus Nivolumab with patients treated with the standard STUPP regimen as a control [71]. This trial reported that mOS was 28.9 and 32.1 months and mPFS was 10.6 and 10.3 months in the Nivolumab and STUPP groups, respectively. In addition, subgroup analysis based on PD‐L1 expression also showed no statistical difference in the survival of patients.

All these three clinical trials showed negative results of ICIs in GBM, and several studies were performed to identify the features of responders to clarify indications of ICIs. TMB, mismatch repair (MMR), and immune checkpoints have been reported as potential biomarkers for ICI treatment in most types of extra‐cranial tumors, not similar in GBM because of low TMB and MMR and immune suppression [72]. Responders of anti‐PD‐1 treatment demonstrated distinct patterns, including tumor clonal evolution, enrichment of mutations in the MAPK pathway, generation of more neoantigens, and lower T cell clonal diversity [73]. An early phase clinical trial manifested that neoadjuvant ICIs promoted the release of immunogenic neoantigens and induced immune responses [74]. More research is warranted on the screening strategies of potential patients and the time sequence of ICIs and others treatments.

#### 3.2.2 Antigenic peptide vaccine

Antigenic peptide vaccines, peptides comprising 8–25 amino acids, are constructed based on the specific sequences of tumor‐specific antigens or tumor‐associated antigens and can induce active antitumor immune responses [75]. EGFRvIII, a specific antigen for GBMs, is expressed in approximately 20%–30% of GBMs [76]. CDX‐110 (Rindopepimut) is an antigenic peptide targeting EGFRvIII. A Phase III clinical trial of CDX‐110 was conducted with the involvement of 745 nGBM patients expressing EGFRvIII [22]. Patients in the experimental group were treated with the standard STUPP regimen combined with adjuvant Rindopepimut [71]. mPFS and mOS were not statistically different in the subgroup of GBM that was evaluated as small residual lesions (size of enhancement lesions on MRI < 2 cm^2^) after radiochemotherapy. For patients with significant residual lesions, mOS was 16.1 and 15.6 months in the Rindopepimut group and the control group, respectively. Common grade 3–4 adverse reactions included thrombocytopenia, malaise, cerebral edema, and seizures. Due to the heterogeneity, EGFRvIII‐negative GBM can progress under Rindopepimut treatment, similar to the results under treatment with other peptide vaccines against a single antigen [75, 77].

Due to the long response time to activate native tumor‐reactive cytotoxic T lymphocytes (CTLs), antitumor immune responses can also be induced by screening the precursor cells of peptide‐specific CTLs already present in peripheral blood and constructing personalized peptide vaccination (PPV) [78]. Previous research on PPV for rGBM indicated that approximately 71% (15/21) of patients developed cellular and humoral immune responses [79] with tolerable adverse reactions [80]. A following phase III clinical trial was carried out by including rGBM patients who were positive for human leukocyte antigen‐A24 [23] and had immune responses to at least 2 peptides of a 12‐peptide library, in which patients in the interventional group were treated with PPV and compared with those treated with placebo. The data demonstrated that mOS was 8.4 and 8.0 months in the interventional and placebo groups, respectively (*p* = 0.621). Subgroup analysis results suggested that in addition to age, body weight, and physical status, SART2‐93 peptide was associated with worse prognoses. Moreover, immune responses were higher in patients without SART2‐93 peptide selection. However, additional immune features of patients should be clarified in the future.

#### 3.2.3 Cellular vaccines

Dendritic cell (DC) vaccines are associated with T‐lymphocyte infiltration in the CNS [81]. Liau et al. [82] conducted a phase III clinical trial of DCVax‐L, a dendritic cell vaccine loaded with autologous tumor lysates, and published the preliminary results. In their trial, patients in the interventional group were subjected to DCVax‐L treatment, while patients in the control group were treated with peripheral blood mononuclear cells [71], and crossover therapy was available for tumor progression, including DCVax‐L and other optimal treatments. Based on the Intention‐to‐Treat cohort (331 cases), 90% of patients receiving DCVax‐L treatment had a mOS of 23.1 months, and approximately 30% had a mOS of up to 46.5 months. In addition, around 2.1% of patients experienced DC vaccine‐related grade 3–4 adverse reactions, including cerebral edema, seizures, nausea, and lymph gland infections. This team published the final results of DCVax‐L in November 2022 [24]. For nGBM patients, mOS was 19.3 and 16.5 months in the DCVax‐L and external control cohorts, respectively (one‐sided *p* = 0.002). For rGBM patients, mOS was 13.2 and 7.8 months in the DCVax‐L and external control groups, respectively (one‐sided *p* < 0.001). Although matching‐adjusted indirect comparison was performed, the historically published external control cohort was used in this study for survival analysis, and the conclusion of the trial of DCVax‐L should be interpreted causally in clinical practice [83]. The chemotaxis of T cells was predicted as a biomarker of DC vaccines, which needed to be confirmed in vivo [84].

#### 3.2.4 Virotherapy

Virotherapy involves viruses that locally and limitedly infect and target renewable cells and performs functions via oncolytic virus and viral vectors carrying therapeutic genes, which has been predicted as an antitumor treatment unaffected by heterogeneity [85, 86, 87, 88].

Retroviruses as vectors carrying the gene encoding herpes simplex virus thymidine kinase (HSV‐tk) are the first to be studied in clinical trials. Thymidine kinases can convert ganciclovir into a nucleoside‐like precursor through phosphorylation to block DNA replication and to induce apoptosis, thereby exerting antitumor immune effects. In a phase III clinical trial of nGBM published by Rainov et al. in 2000 [25], patients in the interventional group were treated with surgical resection and adjuvant radiotherapy combined with HSV‐tk and ganciclovir, for whom HSV‐tk was immediately injected into the residual cavity after surgery, while patients in the control group underwent surgery and radiotherapy. The mOS was 365 days and 354 days in the gene therapy and control groups, respectively. Another study unraveled that adenovirus‐mediated HSV‐tk treatment was more tolerable and was associated with higher immune responses than retrovirus treatment [89]. Westphal et al. [26] then conducted a phase III clinical trial of adenovirus‐mediated HSV‐tk (Sitimagene Ceradenovec, ADV‐tk) by including patients with resectable newly diagnosed supratentorial high‐grade gliomas. The results exhibited a mOS of 497 and 452 days in the experimental and control groups, respectively (*p* = 0.31) and that grade 3–4 adverse reactions in the experimental group were mainly hemiplegia, aphasia, hyponatremia, and seizures.

Toca 511 (Vocimagene Amiretrorepvec) is a nonlytic retroviral replicating vector carrying a codon‐optimized gene encoding the yeast cytosine deaminase enzyme, which can transform the extended‐release precursor drug Toca FC (5‐fluorocytosine, 5‐FC) into 5‐fluorouracil (5‐FU), therefore killing tumor cells and immunosuppressive cells in the microenvironment and inducing antitumor immune responses [90]. Cloughesy et al. [91] performed a phase II/III clinical trial of recurrent high‐grade glioma in 2020, in which patients in the Toca 511 group were injected with Toca 511 into the surgical cavity and administered oral Toca FC 6 weeks after surgery, while patients in the control group were treated with lomustine, TMZ, or bevacizumab. The data showed that mOS was 11.1 and 12.2 months in the Toca 511 and control groups, respectively (*p* = 0.62). Grade 3–4 adverse reactions in the Toca 511 group mainly included aphasia, hemiparesis, and headache.

VB‐111 (Ofranergene Obadenovec) is an adenoviral vector‐based immunotherapy, which carries a hemisynthetic pre‐proendothelin 1 (PPE‐1)‐3x promoter sequence and expresses a chimeric receptor combined with tumor necrosis factor receptor 1 (TNFR1) and Fas. VB‐111 exerts antitumor effects through two following mechanisms: (1) mediating apoptosis and antiangiogenesis in vascular endothelial cells; (2) inducing antitumor responses of T‐lymphocytes [92]. In a phase III clinical trial of rGBM published in 2020 [27], the efficacy of VB‐111 plus bevacizumab was compared with that of bevacizumab monotherapy, which demonstrated that mOS was 6.8 and 7.9 months in the VB‐111 and bevacizumab groups, respectively (*p* = 0.19). In addition, 67.5% of the patients in the VB‐111 group developed grade 3 or higher adverse reactions, including fever, chills, and flu‐like symptoms.

In addition to three of these phase III clinical trials, intra‐tumoral infusion of recombinant polioviruses (PVSRIPO) is neurologically safe and prolongs the survival of patients with rGBM [93]. G47Δ, a triple‐mutated oncolytic herpes simplex virus, is effective and safe for treating GBM according to a phase II clinical trial and has recently been approved in Japan [94]. Furthermore, the cytomegalovirus vaccine (VBI‐1901) also can regulate peripheral CD4+ T cells and benefit the survival of rGBM patients [95]. A preclinical study revealed that the combination of ICIs and oncolytic viruses had complex regulatory effects on both subgroups of T cells and macrophages and showed synergistic curative activity, highlighting the necessity of laboratory study and clinical practice in the future [96].

## 4 SUMMARY AND PERSPECTIVE

### 4.1 Current challenges and perspectives of molecular targeted therapy for GBM

In recent years, the development of cognition and the establishment of the molecular pathology of glioma have provided more options for the clinical treatment of glioma and positively affected glioma treatment. For high‐grade glioma, especially GBM, the classical Stupp protocol prolongs the OS of nGBM patients to 14.6 months, and the subsequent development of TTF increases the OS of GBM patients to approximately 20 months. Nevertheless, it is clear that overall treatment outcomes are disappointing, and standard treatments are lacking for rGBM in particular. Numerous clinical trials of GBM have been conducted over the past 20 years and have almost failed, and even the strategies with significant efficacy in other tumors have been repeatedly frustrated in GBM. This phenomenon is mainly due to the following issues, including the presence of the BBB, the heterogeneity of tumor tissues, and the complexity of the tumor microenvironment. Accordingly, the following attempts may be the key to overcoming these issues: (1) It is highly necessary to develop more effective drug delivery systems, research on which has progressed tremendously and has been detailed in the excellent reviews [97, 98, 99, 100]; (2) for the complex heterogeneity of tumors, more systematic molecular pathological and mechanistic studies are effective. For example, as WHO CNS5 is published, Gene and Protein Nomenclature is formally recommended and verified to be more clinically effective and beneficial. In addition, it lists newly discovered glioma types, uses a method of grading within types, and combines histological and molecular grading, which provides greater clarity in diagnosis and associated treatments. Nonetheless, there still is a lack of molecular diagnosis and precise treatment related to tumor evolution or recurrence at present; (3) the GBM microenvironment is poorly understood relative to GBM cells themselves. GBM has a unique brain tissue environment, such as immune privilege, vast neuronal interactions, and unique brain extracellular matrix components [101, 102]. Therefore, in‐depth research on the role of the microenvironment in which GBM cells reside will advance the diagnostic development of the diagnosis and therapy of GBM. For instance, targeting tumor vessels with bevacizumab improves PFS [15, 32]. In addition, there are positive reports on immunotherapy for GBM [69]. These observations imply a great potential for in‐depth investigation of the GBM microenvironment.

In addition, the history of clinical trials of glioma also suggests that therapeutic strategies targeting a single target or a single pathogenic mechanism often fail due to the high plasticity and redundant survival mechanisms of GBM. Therefore, it is highly imperative to develop combination therapy strategies while developing precision targeted therapies. Moreover, an excellent review has detailed the importance of immune combination therapies [103]. However, current research to develop optimized combination therapies is challenged by the unclear tumor mechanism and numerous combinable post‐permutation therapeutic strategies, which perhaps can be advanced by the development of big data technologies and intelligent experimental platforms [104]. Perhaps the most pressing issue at present is a decrease in efficacy or an enhancement in toxicity when traditional therapies are combined with emerging therapies, as this decrease or enhancement can lead to the failure of clinical trials. For example, TMZ‐induced reductions in PD‐L1 expression may be associated with the treatment failure of nivolumab in rGBM [105]. Due to its immunosuppressive effects, dexamethasone abrogates the efficacy of immunotherapy (particularly PD‐[L]1 treatment) [106]. Therefore, it is extremely necessary to deeply explore the interaction between traditional and emerging therapies before clinical trials.

### 4.2 Current challenges and perspectives of immunotherapy for GBM

The causes of immunotherapy resistance in GBM are complex and may include the following factors: (1) the immunosuppressive microenvironment and overall immune response suppression in patients; (2) heterogeneity during tumor progression, as well as between tumors and patients; (3) low TMB and immunogenicity; (4) the presence of the BBB; (5) pathway redundancy and escape through bypass pathways; (6) a lack of effective biomarkers; and (7) a lack of experimental models [107, 108, 109]. Unlike immunogenic tumors such as lung cancer, GBM has a low level of TMB neoantigens. Although many genetic variants are generated during radiotherapy and TMZ treatment, they cannot effectively transform GBM into a “hot” tumor [110]. Additionally, little is known about the correlation of high levels of TMB and T‐lymphocyte infiltration with responses to immunotherapy [111]. GBM can also lead to T cell trap in the bone marrow, which induces T cell dysfunction [112].

Some phase III clinical trials of GBM elucidated that immunotherapy failed to substantially improve the prognosis of patients. The efficacy of DCVax‐L illustrates the potential of immunotherapy. Furthermore, neoadjuvant therapies, modified drug delivery, researches on new therapeutic targets and combination therapies still have potential in the future [113, 114]. An early clinical trial elaborated that neoadjuvant ICIs enhanced intracranial and systemic antitumor immune responses in rGBM, contributing to survival benefits [74]. Intratumoral drug delivery, nanoparticles, and adjuvant treatment to enhance BBB permeability (such as radiotherapy and focused ultrasound) are promising for overcoming the issue of the BBB [115]. Intratumoral injection of ICIs may prolong the survival of patients with surgically resectable rGBM when compared to bevacizumab treatment in historical cohorts [116]. Combination therapy may be immune microenvironment modulation therapy, other immunotherapy, and radiotherapy [113].

Registered phase III clinical trials of GBM whose results have not been reported are displayed in Table 3. Other immunotherapies, such as chimeric antigen receptor T cells, monoclonal antibodies targeting other immune checkpoints, and vaccine therapy, have also yielded promising results in clinical trials [113, 114].

**Table 3:** Unpublished and registered phase III clinical trials of immunotherapy for GBM in registration.

Immunotherapy	ID	Beginning year	Population	Intervention	Status
ICIs	NCT04396860	2020	nGBM, MGMT promoter unmethylation	RT+NIVO+IPI	Suspended
Peptide vaccine	NCT03149003	2017	rGBM	DSP‐7888	Active, not recruiting
Cellular vaccine	NCT02546102	2015	nGBM, IDH‐WT	ICT‐107	Suspended
Cellular vaccine	NCT03548571	2018	nGBM, IDH‐WT, MGMT promoter unmethylation	DCs transfected with mRNA from autologous tumor stem cells, survivin and hTERT	Active, not recruiting
Cellular vaccine	NCT04277221	2019	rGBM	Autologous dendritic cell/tumor antigen, ADCTA	Recruiting
Cellular vaccine	NCT05100641	2022	nGBM	AV‐GBM‐1	Not yet recruiting
Immune cell	NCT00807027	2008	nGBM	Activated T lymphocyte	Completed
Cellular vaccine+Immune cell	NCT01759810	2012	rGBM	Dendritic vaccine, allogeneic hematopoietic stem cells, cytotoxic lymphocytes	Enrolling by invitation

Abbreviations: DC, dendritic cell; hTERT, telomerase reverse transcriptase in humans; ICIs, immune checkpoint inhibitors; MGMT, *O*‐6‐methylguanine‐DNA methyltransferase; nGBM, newly diagnosed glioblastoma; rGBM, recurrent glioblastoma.

Due to the heterogeneity of GBM, monotherapy is difficult to exert antitumor effects in GBM, and dynamic changes of biomarker‐based treatments are necessary [117].

## AUTHOR CONTRIBUTIONS

**Yuekun Wang**: Writing—original draft (equal); writing—review & editing (equal). **Shenglan Li**: Writing—original draft (equal); writing—review & editing (equal). **Yichen Peng**: Writing—review & editing (equal). **Wenbin Ma**: Conceptualization (equal); writing—review & editing (equal). **Yu Wang**: Conceptualization (lead); writing—review & editing (equal). **Wenbin Li**: Conceptualization (lead); writing—review & editing (lead).

## CONFLICT OF INTEREST STATEMENT

Professor Wenbin Li is the member of the *Cancer Innovation* Editorial Board. To minimize bias, he was excluded from all editorial decision‐making related to the acceptance of this article for publication. The remaining authors declare no conflict of interest.

## ETHICS STATEMENT

Not applicable.

## INFORMED CONSENT

Not applicable.

## Data Availability

The authors have nothing to report.
